# Delivering New Data: Local Traffic Pollution and Pregnancy Outcomes

**DOI:** 10.1289/ehp.117-a505a

**Published:** 2009-11

**Authors:** Julia R. Barrett

**Affiliations:** **Julia R. Barrett**, MS, ELS, a Madison, Wisconsin–based science writer and editor, has written for *EHP* since 1996. She is a member of the National Association of Science Writers and the Board of Editors in the Life Sciences

Up to 35% of preterm births are due to preeclampsia, a complication in 2–8% of pregnancies that is characterized by maternal high blood pressure, edema, protein in the urine, and abnormal liver function. Exposure to certain air pollutants is associated with prematurity and may also be linked with preeclampsia. A new study is the first to home in on specific components of air pollution—those generated by traffic—as being associated with preeclampsia and further supports their role in preterm birth **[*EHP*** **117:1773–1779; Wu et al.]**.

Preeclampsia, which resolves only with delivery of the baby, can cause maternal illness and death, intrauterine growth restriction, preterm birth, and infant death. Each year more than half a million infants in the United States are born prematurely (at less than 37 weeks’ gestation) and consequently face increased risks for developmental delays, lifelong health problems, and neonatal death. These challenges are particularly severe for infants born prior to 30 weeks’ gestation.

The study was based upon 81,186 singleton births that occurred during 1997–2006 at four Southern California hospitals within the same health care system. The system’s database provided information on the mothers’ demographic characteristics, medical history, and pregnancy complications; their home address at the time they gave birth; and their infants’ gestational age, sex, and birth weight.

Traffic pollution generated within a 3-km radius of each mother’s residence was estimated using a comprehensive dispersion model that incorporated meteorologic variables (such as atmospheric stability and wind), roadway geometry, traffic counts, and vehicle emission factors. The exhaust components nitrogen oxides (NO_x_) and particulate matter smaller than 2.5 μm (PM_2.5_) served as surrogates for local traffic pollution in the model.

The researchers estimated average exposures over the entire pregnancy at approximately 7 ppb for NO_x_ and 2 μg/m^3^ for PM_2.5_. After accounting for other factors that might be related to preeclampsia and exposure, the authors estimated that pregnant women in the highest quartile of PM_2.5_ exposure had a 42% increased relative risk of preeclampsia compared with women in the lowest quartile, and those in the highest quartiles of NO_x_ and PM_2.5_ exposure had 128% and 81% higher relative risk than women in the lowest quartiles, respectively, for delivery at less than 30 weeks’ gestation. The sophisticated dispersion model and detailed individual clinical data are particular strengths of the study, but the findings are limited by information that was not available, such as workplace exposures, changes in residence during pregnancy, and maternal smoking.

The researchers speculate that the toxic mechanisms described in air pollution studies of respiratory and cardiovascular diseases—specifically, oxidative stress and a generalized inflammatory response—might also partly explain preterm delivery and preeclampsia. They emphasize that the current study does not specifically indict NO_x_ and PM_2.5_, although the results support a connection between traffic-related air pollution and adverse reproductive outcomes.

